# Geomicrobiological Features of Ferruginous Sediments from Lake Towuti, Indonesia

**DOI:** 10.3389/fmicb.2016.01007

**Published:** 2016-06-30

**Authors:** Aurèle Vuillemin, André Friese, Mashal Alawi, Cynthia Henny, Sulung Nomosatryo, Dirk Wagner, Sean A. Crowe, Jens Kallmeyer

**Affiliations:** ^1^GFZ German Research Centre for Geosciences, Helmholtz Centre Potsdam, Section 5.3 Geomicrobiology, PotsdamGermany; ^2^Research Center for Limnology, Indonesian Institute of SciencesCibinong, Indonesia; ^3^Department of Microbiology and Immunology, University of British ColumbiaVancouver, BC, Canada; ^4^Department of Earth, Ocean, and Atmospheric Sciences, University of British ColumbiaVancouver, BC, Canada

**Keywords:** bottom waters, iron-rich sediment, sedimentary microbes, extracellular DNA, sulfate reduction, iron reduction, Lake Towuti

## Abstract

Lake Towuti is a tectonic basin, surrounded by ultramafic rocks. Lateritic soils form through weathering and deliver abundant iron (oxy)hydroxides but very little sulfate to the lake and its sediment. To characterize the sediment biogeochemistry, we collected cores at three sites with increasing water depth and decreasing bottom water oxygen concentrations. Microbial cell densities were highest at the shallow site—a feature we attribute to the availability of labile organic matter (OM) and the higher abundance of electron acceptors due to oxic bottom water conditions. At the two other sites, OM degradation and reduction processes below the oxycline led to partial electron acceptor depletion. Genetic information preserved in the sediment as extracellular DNA (eDNA) provided information on aerobic and anaerobic heterotrophs related to *Nitrospirae*, *Chloroflexi*, and *Thermoplasmatales*. These taxa apparently played a significant role in the degradation of sinking OM. However, eDNA concentrations rapidly decreased with core depth. Despite very low sulfate concentrations, sulfate-reducing bacteria were present and viable in sediments at all three sites, as confirmed by measurement of potential sulfate reduction rates. Microbial community fingerprinting supported the presence of taxa related to *Deltaproteobacteria* and *Firmicutes* with demonstrated capacity for iron and sulfate reduction. Concomitantly, sequences of *Ruminococcaceae*, *Clostridiales*, and *Methanomicrobiales* indicated potential for fermentative hydrogen and methane production. Such first insights into ferruginous sediments showed that microbial populations perform successive metabolisms related to sulfur, iron, and methane. In theory, iron reduction could reoxidize reduced sulfur compounds and desorb OM from iron minerals to allow remineralization to methane. Overall, we found that biogeochemical processes in the sediments can be linked to redox differences in the bottom waters of the three sites, like oxidant concentrations and the supply of labile OM. At the scale of the lacustrine record, our geomicrobiological study should provide a means to link the extant subsurface biosphere to past environments.

## Introduction

**FIGURE 1:**
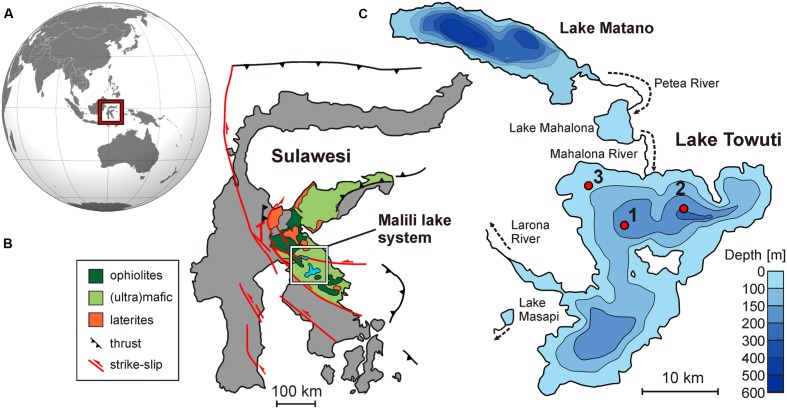
**Lake Towuti location and settings. (A)** Map of Asia and Oceania displaying the location of Sulawesi Island. **(B)** Map of Sulawesi illustrating the geological context of the Malili lake system (modified after Calvert and Hall, 2007). **(C)** Bathymetric map of Lake Matano and Lake Towuti (modified after Herder et al., 2006) displaying the three sites at which gravity cores were retrieved. Sites 1–3 correspond respectively to water depths of 153, 200, and 60 m, with oxygenation conditions at the water–sediment interface decreasing with water depth.

Lake Towuti is a tropical 200 m deep tectonic lake seated in ophiolitic rocks and surrounded by lateritic soils (Lehmusluoto et al., 1995; Russell and Bijaksana, 2012). It is part of the Malili Lakes system, comprising several interconnected lakes, including Lake Matano, the 10th deepest lake in the world (~600 m). Its location on Sulawesi, Indonesia (**Figure 1A**) renders Lake Towuti’s sediments prime recorders of paleoclimatic changes in the tropical Western Pacific warm pool (Russell et al., 2014). The tropical climate and the lateritic weathering of the (ultra)mafic catchment of the Malili Lakes system (**Figure 1B**) cause a strong flux of iron to the lake. Surrounding lateritic soils are typically related to limonite types, with mostly goethite (α-FeOOH) and ferrihydrite (Fe_2_O_3_⋅0.5H_2_O) transported to the basin (Crowe et al., 2004; Golightly, 2010) as well as some hematite (Fe_2_O_3_) and detrital magnetite (Fe_3_O_4_). High iron fluxes to the lake may exert a decisive constraint on bioavailable phosphorus in the epilimnion as it is scavenged by iron (hydr)oxides, likely driving Lake Towuti’s water column toward severely nutrient-limited conditions. However, anoxia in stratified water column can lead to iron reduction and partial release of adsorbed P into the water at the oxycline and below (Zegeye et al., 2012). Biogeochemical and microbiological data gathered from nearby Lake Matano reveal persistent anoxia in the deeper part of Lake Matano’s water column (Crowe et al., 2008b; Jones et al., 2011) with organic matter (OM) degradation through methanogenesis (Katsev et al., 2010; Crowe et al., 2011). Although Lake Towuti is anoxic at greater depths as well, it is less deep and can mix periodically (Haffner et al., 2001), presumably causing transient bottom water oxygenation (Costa et al., 2015).

Once buried, ferruginous sediments likely support microbial communities, which can utilize a range of metalliferous substrates (Crowe et al., 2007). Although microbial activity decreases dramatically below the water–sediment interface and with increasing sediment depth (Kallmeyer et al., 2012), even this low activity can have an appreciable impact on both sediment composition and biogeochemical cycles over long-time periods (Berner, 1980; Freudenthal et al., 2001; Horsfield and Kieft, 2007). In addition, iron minerals are also suspected to strongly adsorb DNA (Cecchio et al., 2005; He et al., 2008). Upon cell lysis, nucleic acids are released into the surrounding water and sediment and partitioned between sorption to reactive Fe surfaces (Pietramellara et al., 2008; Ceccherini et al., 2009) and uptake or degradation via microbial metabolisms (Corinaldesi et al., 2007, 2008). Binding to metal oxides and colloids (Ceccherini et al., 2009; Cleaves et al., 2011) could result in preservation and persistence of extracellular DNA (eDNA) in the lacustrine record (Pietramellara et al., 2008), providing a valuable archive of genetic information (Corinaldesi et al., 2011). However, since metal-reducing bacteria have the capacity to solubilize structural Fe and utilize adsorbed nutrients (Dong et al., 2003; Crowe et al., 2007), the sediment-bound eDNA should not be totally recalcitrant (Baldwin, 2013 and references therein) and could serve as a labile organic substrate for sedimentary microbes (Corinaldesi et al., 2007). Its concentrations should then depend on the complex interplay between these processes (Dell’Anno and Corinaldesi, 2004). Altogether, Lake Towuti provides the opportunity to examine microbial populations in an iron-dominated and sulfate-poor ecosystem with dynamic redox conditions and infer sorption and diagenetic processes arising from subsurface microbial activity.

In order to investigate the relationship between biogeo chemistry and microbial processes in these iron-rich anoxic sediments, we retrieved short sediment cores from Lake Towuti in 2013 and 2014 at three different depths (**Figure 1C**). The sediment was analyzed for pore water geochemistry, total cell counts and potential sulfate reduction rates (SRR) as well as eDNA and intracellular DNA (iDNA). These data provide a background for geomicrobiological and biogeochemical analysis of the long (>100 m) drill cores that were retrieved during the ICDP (International Continental Scientific Drilling Program) Towuti drilling campaign in spring/summer 2015.

## Materials and Methods

### Site Description

**FIGURE 2:**
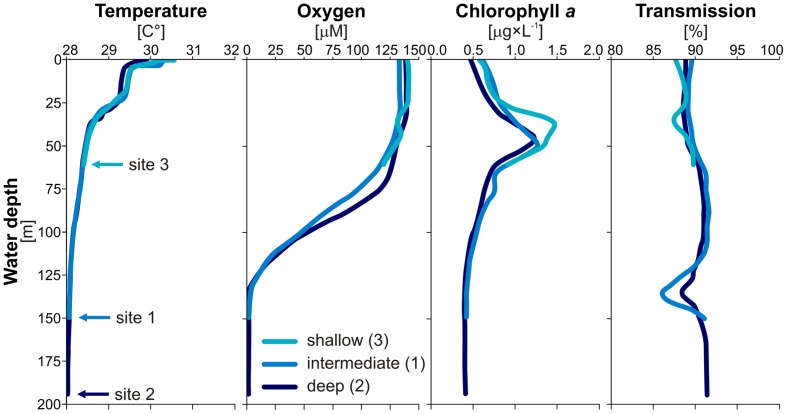
**Water column profiles.** CTD casts for temperatures (°C), oxygen concentrations (μM), chlorophyll *a* (μg × L^-1^), and light transmission [%] measured in the water column of Lake Towuti at each of the three sites. Results show that Lake Towuti is weakly thermally stratified with an oxycline occurring in between 90 and 130 m depth. Chlorophyll *a* peaks up at 50 m depth, while the presence of suspended particles can be inferred from the transmission decrease at 130 m depth.

Lake Towuti (2.5°S, 121°E) is the largest lake within the Malili Lake System, a chain of five tectonic lakes in Sulawesi, Indonesia (Lehmusluoto et al., 1995). It has a surface area of 560 km^2^ and a maximum water depth of 203 m (Haffner et al., 2001). Lake Towuti has a tropical humid climate, with an annual average air temperature of 26°C and little variations in monthly temperatures. Precipitation averages 2540 mm year^-1^, with no distinct dry season (Aldrian and Susanto, 2003). The Mahalona River, which is the main inflow to the north, drains the extensive catchments of Lakes Matano and Mahalona, while the Larona River constitutes the only outflow to the west (**Figure 1C**). Lake Towuti is weakly conductive (210 μS cm^-1^) and circumneutral (pH ~7.8) with a chemistry dominated by Mg and HCO_3_^-^ (Lehmusluoto et al., 1995). Surface water temperatures are commonly 28–29°C, with weak thermal stratification of the water column (i.e., 28–31°C; **Figure 2**). Unlike upstream Lake Matano, which is permanently stratified and anoxic below 110 m water depth due to its great depth (600 m) and steep slopes (Crowe et al., 2008b; Katsev et al., 2010), the entire water column of Lake Towuti is reported to mix at least occasionally (Haffner et al., 2001; Costa et al., 2015). Lake Towuti is usually oxygen-depleted below 130 m depth (**Figure 2**). As a result of sedimentary P trapping due to large amounts of iron oxides derived from lateritic soils being transported into the basin (Zegeye et al., 2012), productivity in the water column is expected to be limited (Bramburger et al., 2008).

### Sample Processing

Water temperature, oxygen concentration, light fluorescence reemitted by chlorophyll *a* (Leeuw et al., 2013), and light transmission profiles were collected on site using a submersible conductivity-temperature-depth probe (CTD; Sea-Bird, SBE-19; Sea-Bird Electronics, Bellevue, WA, USA). Temperature profiles showed that the water column of Lake Towuti is indeed weakly stratified while oxygen concentrations indicated that the water–sediment interface at the bottom of Lake Towuti is variably oxygenated depending on water depth, with anoxia in waters below 130 m (**Figure 2**). Several sediment cores (<0.5 m) estimated to cover ca. 1750 years of sedimentation history (Tamuntuan et al., 2015; Vogel et al., 2015) were retrieved at three sites with increasing water depth (60, 153, and 200 m; **Figure 1C**) and different oxidation states in overlying waters (**Figure 2**), the intermediate site (153 m depth) was chosen to be the main site of the ICDP Towuti Drilling Project. The cores were sampled for pore water geochemistry, total cell counts, potential SRR and small subunit (16S) rRNA gene fingerprinting analyses.

Pore water sampling was carried out on site under anoxic conditions, using a glove bag flushed with nitrogen gas in order to prevent oxidation of the sediment. Using an aseptic spatula, we sectioned the sediment cores in 0.5, 1, and 2 cm resolution for the upper 1, 1–10, and below 10 cm, respectively. Sediment samples were transferred into 50 mL centrifuge tubes and Rhizon Pore Water Samplers (Rhizon CSS, Rhizosphere research products, Dolderstraat, Netherlands) were inserted into the sediment through a hole in the lid. For each depth interval, 10 mL of pore water was collected in syringes, filtered through 0.2 μm pore size cellulose acetate membrane syringe filters (Minisart, Sartorius Stedim Biotech) to remove all particles and most microorganisms, transferred into 2 mL twist top vials and stored at room temperature for analysis in the home lab.

Samples for total cell counts were preserved in a 0.2 μm filtered fixative solution of lake water amended with formalin (final concentration 2%). For each sample, 2 cm^3^ of sediment was retrieved with a sterile 3 mL cut-off syringe, placed in 15 mL centrifuge tube filled with 8 mL of fixative solution and shaken for complete homogenization. The tubes were sealed and stored at 4°C until analysis in the home lab.

For nucleic acid analyses, entire short cores were sampled inside the nitrogen-filled glove bag. Using an aseptic spatula, we sectioned the cores into 1, 2, and 5 cm intervals for the first 10 cm, between 10 and 20 cm and below 20 cm depth, respectively. Samples were packed into gas-tight aluminum foil bags, flushed with nitrogen gas and heat-sealed to keep them under anoxic conditions. Until DNA extraction in the home lab the samples were stored at room temperature. Since lake temperatures are ca. 28°C throughout the year, we considered this storage appropriate to minimize cell lysis or potential shifts in the microbial assemblage due to refrigeration. Freezing was not an option as it would cause cell lysis and therefore artificially increase the eDNA pool.

Samples for potential SRR measurements were collected in triplicate using glass barrels with approximately 5 cm^3^ volume. The glass barrels were used to retrieve small sub-cores from the undisturbed inner part of the core, sealed with a butyl rubber stopper and stored in sealed, nitrogen-flushed gas-tight aluminum foil bags at room temperature until incubation in the home lab.

### Laboratory Procedures

#### Total Organic Carbon and Total Nitrogen

Total nitrogen (TN) and total carbon (TC) were measured, using a Vario EL III CHNOS elemental analyzer (Elementar Analysensysteme, Hanau, Germany). Each sample tin cup was loaded with 8–9 mg of freeze-dried, powdered sediment plus a spatula tip of tungsten oxide (WO_3_) to enhance the combustion, and then combusted at 1150°C. Detection limit is below 0.1%. TC results were used to calibrate volumes of sample required for total organic carbon (TOC) measurements. For TOC analysis, between 30 and 70 mg of sediment powder were loaded into a crucible and burnt at 580°C (sensitive carbon T° for non-calcareous samples), 850 and 1150°C (intensive carbon T° for calcareous samples) using a Vario MAX cube elemental macro analyzer (Elementar Analysensysteme), equipped with the soliTIC option for the determination of the total organically bound and inorganic carbon in the sample. Pretreatment of samples with HCl (5%), as is normally done for standard elemental analyzers (Byers et al., 1978) is not needed with this device. TOC and TN were recalculated to the content of the whole sample to present results in dry mass %. TOC and TN values were then corrected by their respective atomic weights (i.e., 14/12) and used to calculate molar C_org_/N ratios.

#### Iron Spectrophotometry

Dissolved iron concentrations in pore water were measured in the field via spectrophotometry (Viollier et al., 2000). Directly after pore water retrieval, we aliquoted 1 mL of pore water sample to 1.6 mL Rotilabo single-use cells (Carl Roth, Karlsruhe, Germany) and stabilized dissolved Fe^2+^ by adding 100 μL of Ferrozine Iron Reagent (Sigma-Aldrich Chemie, Munich, Germany). To avoid oxidation during handling, all sample handling was carried out inside the nitrogen-filled glove bag. Absorbance of the colored solution was measured at 562 nm with a DR 3900 spectrophotometer (Hach, Düsseldorf, Germany). Detection limit of the method is 0.25 μM.

#### Ion Chromatography

Cation and anion concentrations in pore water samples were analyzed using an ion chromatography (IC) system (Sykam Chromatographie, Fürstenfeldbruck, Germany). Injected sample volume was 50 μL for both anions and cations. For cations, the IC system consisted of a S5300 sample injector (Sykam), a 4.6 × 125 mm Reprosil CAT column (Dr. Maisch HPLC, Ammerbuch-Entringen, Germany) and a S3115 conductivity detector (Sykam). The eluent was 2.5 mM HNO_3_. Flow rate was set at 1 mL min^-1^ and column oven temperature at 45°C. A Cation Multi-Element Standard (Carl Roth) was diluted five times for calibration. Based on a respective signal-to-noise (S/N) ratio of 3 and 10 (Schibler et al., 2007), the detection and quantification limits were calculated for each ion and are as follows: Li^+^ (10.5 μM; 63.5 μM), Na^+^ (5.8 μM; 35 μM), K^+^ (9.1 μM; 54.7 μM), Mg^2+^ (9.6 μM; 44.6 μM), Ca^2+^ (8.3 μM; 38.5 μM) and NH_4_^+^ (11.3 μM; 67.6 μM). Samples were measured in triplicates. During each run, nine standards were measured to check for drift. Reproducibility was always better than 5% for each ion. Concentrations are given as average values based on triplicates, error bars are one standard deviation.

Anions were analyzed by suppressed IC using a SeQuant SAMS anion IC suppressor (EMD Millipore, Billerica, Massachusetts), a S5200 sample injector, a 3.0 × 250 mm lithocholic acid (LCA) 14 column and a S3115 conductivity detector (all Sykam). The eluent was 5 mM Na_2_CO_3_ with 20 mg L^-1^ 4-hydroxybenzonitrile and 0.2% methanol. Flow rate was set to 1 mL min^-1^ and column oven temperature to 50°C. A multi-element anion standard (Sykam) containing F^-^ (263.2 μM), Cl^-^ (564.1 μM), Br^-^ (250.3 μM), NO_2_^-^ (434.7 μM), PO_4_^3-^ (210.6 μM), NO_3_^-^ (806.4 μM), and SO_4_^2-^ (520.5 μM) was diluted 10 times and measured every 10 samples. Respective minimum detection (S/N = 3) and quantification limits (S/N = 10) are as follows: F^-^ (2.3 μM; 13.3 μM), Cl^-^ (5.7 μM; 16.17 μM), NO_2_^-^ (4.1 μM; 14.14 μM), Br^-^ (3 μM; 10.2 μM), NO_3_^-^ (2.8 μM; 9.3 μM), PO_4_^3-^ (4.3 μM; 14.3 μM) and SO_4_^2-^ (2 μM; 8.4 μM). The reproducibility was always better than 3% for each ion.

For ammonium (NH_4_^+^) concentrations, the cation IC system was converted to a flow injection analysis system (Hall and Aller, 1992) using a second pump and a dialysis cell (Skalar Products, Breda, Netherlands) instead of a separation column. First, NH_4_^+^ is quantitatively converted into NH_3_ by injecting the sample into an alkaline release solution containing 53.75 g L^-1^ citric acid and 70 g L^-1^ sodium hydroxide. The NH_3_ diffuses through a Teflon membrane into a capture solution made of 1 g L^-1^ boric acid and reacts to form ammonium borate. The associated change in conductivity is measured with a conductivity detector. The flow rate for release and capture solution was 0.1 and 0.3 mL min^-1^, respectively. For 100 μL injection volume, the minimum detection (S/N = 3) and quantification limits (S/N = 10) are 5 and 12.2 μM, respectively. Samples were measured in triplicates, the reproducibility was always below 10%.

#### Potential Sulfate Reduction Rate Quantification

Potential SRR quantification was performed according to Jørgensen (1978) and Kallmeyer et al. (2004). In brief, 3 kBq of ^35^SO_4_^2-^ radiotracer were injected into each sediment plug and incubated for 24 h in the dark close to situ temperature (ca. 30°C). Incubated sediments were transferred into 10 mL of 20% (w/v) zinc acetate and homogenized to precipitate free sulfide as zinc sulfide and stop microbial activity. The sample slurry was centrifuged (4000 × *g*, 20 min) and 1 mL of supernatant transferred into a 7 mL scintillation vial with 4 mL of Ultima Gold Cocktail (Perkin Elmer, Waltham, MA, USA) for quantification of unreacted ^35^SO_4_^2-^. The remaining sediment was flushed out with 20 mL dimethylformamide into a four-neck glass flask. Non-radioactive zinc sulfide was added as a carrier, followed by injection of 8 mL of 6 N HCl and 16 mL of 1 M CrCl_2_ solution and stirring of the sample for 2 h under a constant flow of nitrogen gas. The released H_2_S was guided through a trap filled with 7 mL of 0.1 M of a buffered citric acid/Na-citrate solution to trap ^35^SO_4_^2-^-containing aerosols. A second trap filled with 7 mL of 5% (w/v) zinc acetate was used to precipitate H_2_S as zinc sulfide. At the end of the distillation, the zinc acetate solution was quantitatively transferred into a scintillation vial with 8 mL of Ultima Gold Cocktail (Perkin Elmer). Radioactivity of radiolabeled sulfide was quantified using a Tri Carb 2500 TR liquid scintillation counter (Packard Instruments, Meriden, CT, USA).

#### Total Cell Counts

Cell counts were performed using a modification of the procedure of Kallmeyer et al. (2008). In brief, 50 μL of sediment slurry were mixed with 50 μL of detergent mix (i.e., 36.8 g L^-1^ Na_2_ EDTA × 2 H_2_O, 22.3 g L^-1^ Na-pyrophosphate × 10 H_2_O and 5 mL TWEEN 80), 50 μL of methanol and 350 μL of ultrapure H_2_O. This mixture was vortexed for 30 min and sonicated 4 × 1 s at 10% amplitude, using a Sonopuls HD 3200 ultrasonic homogenizer equipped with a microtip probe (Bandelin Electronic, Berlin, Germany). Fifty microliters of this solution were mixed with 5 μL of 1% hydrogen fluoride (HF) to dissolve fine mineral particles (Morono et al., 2009). After 10 minutes, the solution was filtered onto black 0.2 μm polycarbonate track-etched membrane filters (Ø 25 mm Cyclopore, Whatman International Ltd, Maidstone, UK). Cells were stained with SYBR Green I (Molecular Probes Inc., Eugene, OR, USA), mounted and counted by epifluorescence microscopy (Leica DM2000 microscope, 100× magnification) with a blue filter set (Leica Filter Cube F1/RH, excitation filter λ = 490/15 to 560/25 nm, suppression filter at λ = 525/20 to 605/30 nm) and non-fluorescent immersion oil (Leica type F oil, n_e_^23^ = 1.518, v_e_ = 46).

#### Intracellular and Extracellular DNA Extraction and Quantification

The procedure of Alawi et al. (2014) was applied to extract eDNA and iDNA separately from single sediment samples. All extractions were performed in duplicates along with a negative control. In brief, we mixed 1.0 g of fresh sediment with 0.2 g of acid washed polyvinylpolypyrrolidone (PVPP) and 2.5 mL of 0.1 M sodium phosphate buffer (Na-P-buffer). The sample slurry was centrifuged and the supernatant decanted off twice for a final volume of 7.5 mL. The supernatant was centrifuged for 45 min at 4700 × *g* to separate the iDNA (i.e., cell pellet, also containing viral particles) from the eDNA (i.e., supernatant). To avoid any DNA adsorption onto the rubber, we used 10 mL syringes without rubber and filtered the supernatant through 0.2 μm cellulose acetate syringe filters (Sartorius, Göttingen, Germany) to remove any residual PVPP. The filtrate was mixed with three times its volume of 6 M guanidine hydrochloride. Sixty microliters of silica particles were added to adsorb eDNA (Boom et al., 1990). After centrifugation, the supernatant was discarded and the eDNA-containing silica pellet rinsed in 150 μL of absolute ethanol and Tris-EDTA in equal amounts and centrifuged twice for complete drying. To desorb the eDNA, 150 μL of Tris-EDTA buffer (1 mM) were added to the silica pellet, vortexed and centrifuged. The final eDNA-containing supernatant was decanted off and stored. The desorption step was repeated to reach a final volume of 300 μL of eDNA extract.

The iDNA extraction was performed using the Mobio PowerSoil DNA extraction and isolation kit. The content of the PowerBead Tube with 60 μL of solution C1 and 500 μL of Na-P-buffer were added to a 15 mL centrifuge tube containing the cell pellet. The mixture was vortexed, sonicated for 6 min, heated twice for 5 min at 70°C to lyse cells and centrifuged for 15 min at 4700 × *g*. The supernatant was processed in the same as the eDNA fraction adding 50 μL of silica particles. Final iDNA elution volume was 100 μL.

DNA concentrations of all samples were measured using a Qubit 2.0 fluorometer (Invitrogen, Carlsbad, CA, USA) with 10 μL of DNA template, 1 μL of reagent and 190 μL of buffer solution. The Qubit fluorescent dye targets DNA specifically, as compared to UV absorbance techniques which measure any compound absorbing at 260 nm (i.e., DNA, RNA, protein, free nucleotide, and excess salt). Measurements were performed in duplicates and the results are given as the average, error bars are one standard deviation.

#### Polymerase Chain Reaction

DNA extracts were purified following the Mobio PowerSoil experienced user protocol (www.mobio.com) and eluted in 100 μL for eDNA and 65 μL for iDNA. Purified DNA extracts were diluted 20 and 10 times for eDNA and iDNA, respectively, and used as templates in polymerase chain reaction (PCR) amplifications. PCR was performed with 2.5 μL of DNA template, 12.5 μL of MangoMix (Bioline, Life Science Company, London, UK), 0.5 μmol L^-1^ of each of the primers, 1 μL of MgCl_2_ (25 mM) and 8 μL of Ultra-pure 18.2 Mω PCR Water (Bioline). Negative controls were added to all PCR sets with 2.5 μL of molecular grade water as template. Amplifications of the bacterial small subunit (16S) rRNA gene were performed using the bacterial universal primer pair GC-Uni331F (5′-CGC CCG CCG CGC GCG GCG GGC GGG GCG GGG GCA CGG GGG GTC CTA CGG GAG GCA GCA GT-3′) and Eub797R (5′-GGA CTA CCA GGG TAT CTA ATC CTG TT-3′; Brands et al., 2009). PCR cycles were run as follows: 30 cycles of 95°C for 1 min, 57°C for 1 min, and 72°C for 1 min, with a final extension step of 10 min at 72°C. Amplifications of the archaeal 16S rRNA gene were performed using the archaeal universal primer pair GC-UA751F (5′-CGC CCG CCG CGC GCG GCG GGC GGG GCG GGG GCA CGG GGG GCC GAC GGT GAG RGR YGA A-3′) and UA1204R (5′-TTM GGG GCA TRC IKA CCT-3′; Baker and Cowan, 2004). PCR cycles were run as follows: 35 cycles of 95°C for 1.5 min, 60°C for 1.5 min, and 72°C for 1.5 min, with a final extension step of 10 min at 72 °C. For denaturing gradient gel electrophoresis (DGGE), 120 μL archaeal PCR product were concentrated to 25 μL using the Hi Yield PCR Clean-up and Gel-Extraction Kit (SLG Südlabor, München, Germany).

#### Denaturing Gradient Gel Electrophoresis Analysis

Eight percent polyacrylamide gels were prepared with a linear gradient from 35 to 60% of the denaturants urea and formamide. The 100% gradient solution was prepared using 1 mL of TAE (50×), 21 g urea, 20 mL of formamide, 10 mL of 40% acrylamide/bis-acrylamide, and molecular grade water for a final volume of 50 mL. The 0% gradient solution was prepared without urea or formamide. Gels were prepared with 17.5 mL of each solution, plus 50 μL ammonium persulfate solution (APS) and 50 μL tetramethylethylenediamine (TEMED) for polymerization. Twenty microliters of PCR product and 20 μL of blue stain were loaded for each sample. Ten microliters of standard (i.e., 13 previously selected bands) with 10 μL of blue stain were loaded in the center and on each side of the gels to reference band positions. Electrophoresis was run at 100 V for 16 h in 1× TAE buffer at 60°C. Gels were stained for 30 min in 2.5 μL of SYBR Gold nucleic acid gel stain (10,000 time concentrates, Invitrogen) diluted in 25 mL 1× TAE buffer. Gels were photographed under UV light. The Strati-Signal software (Vuillemin et al., 2011; Ndiaye et al., 2012) was then used to analyze DGGE gel pictures. Band numbers, intensity values and pattern lengths were extracted (Supplementary Material) to calculate Shannon indices. The Shannon index is calculated as follows: Shannon = -Σ (*ni/N*) × log (*ni/N*), where *ni* is the height of each peak and *N* the sum of all peak heights extracted from red green blue (RGB) intensity curves of each sample (Boon et al., 2001; Fromin et al., 2002).

#### Band Sequencing and Phylogenetic Analysis

In total, 131 bands were excised from DGGE gels under UV light. Gel bands were eluted in 20 μL Mω PCR Water (Bioline) and used as templates in PCR mixtures as described above without GC-clamp on the forward primers. PCR cycles were run as follows: three cycles of 95°C for 1 min, 57°C for 1 min, and 72°C for 1 min followed by 27 cycles of 95°C for 30 s, 57°C for 30 s, and 72°C for 30 s with a final extension step of 10 min at 72°C for Bacteria, and 35 cycles of 95°C for 50 s, 58°C for 50 s, and 72°C for 50 s with a final extension step of 10 min at 72°C for Archaea. PCR products were run on 1% agarose gels and targeted DNA bands excised under UV light to remove residual dimers. DNA bands were purified using the Hi Yield PCR Gel-Extraction Kit (SLG Südlabor) with final elution in 25 μL of Ultra-pure 18.2 Mω PCR Water (Bioline). DNA concentrations were checked on a NanoPhotometer P330 (Implen, München, Germany) and DNA extracts sent to GATC Biotech AG (European Custom Sequencing Centre, Köln, Germany) for Sanger sequencing using primers Eub797R and UA751F. Sequences were aligned on Sequencher v. 5.1 (Gene Codes Corporation, Ann Arbor, MI, USA) and primers selectively cut off. Chimeras were detected using the online program Bellerophon (Huber et al., 2004). All DGGE sequences have been deposited in the GenBank database under accession numbers KR091588 to KR091718.

The SINA online v.1.2.11 (Pruesse et al., 2007, 2012) was used to align, search and classify our sequences. Closest match sequences were downloaded from the SILVA database as taxonomic references, uploaded on the ARB platform and plotted into phylogenetic trees using the maximum-likelihood method with the RAxML algorithm with advanced bootstrap refinement from 100 replicates (Ludwig et al., 2004). DGGE fragment sequences were added to the RAxML tree using the bacteria positional variability by parsimony algorithm. Two separate phylogenetic trees were plotted for Bacteria and Archaea with iDNA and eDNA sequences together (Supplementary Material). Sequences from representative taxa were then selected to build one single tree based on 15 eDNA and 15 iDNA fragments (**Figure 4**).

## Results

### Water Column

CTD water column profiles (**Figure 2**) obtained at each site provided evidence for weak thermal stratification occurring around 25 m water depth with a corresponding drop of 1.5°C. Below this depth, water column temperatures did not vary significantly, with less than 1°C decrease in the subsequent 175 m. Oxygen profiles indicated that the water column is fully oxic (250 μM) down to 60 m depth where oxygen concentration declines gradually over the following 70 m, the deepest part of the water column is anoxic. Thus, the water–sediment interface could be considered oxic at the shallow site (i.e., 60 m depth) and anoxic at the intermediate (i.e., 153 m depth) and deep sites (i.e., 200 m depth). Chlorophyll *a* fluorescence profiles showed that phytoplankton biomass is highest at 50 m water depth. Transmission profiles revealed negative peaks around 50 and 130 m depth indicating higher concentrations of suspended particles in these depth intervals.

### Pore Water Geochemistry

**FIGURE 3:**
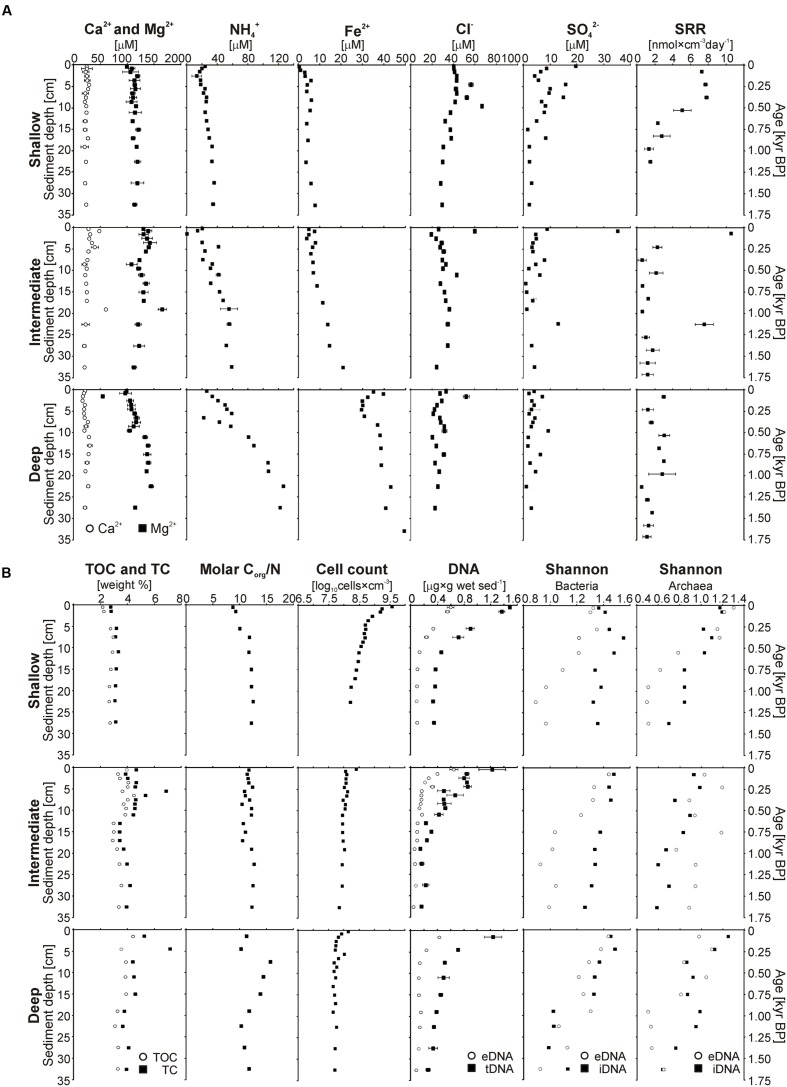
**Multiple profiles established on short sediment cores.** For consistency, results for the three different sites are displayed vertically according to their water depths. **(A)** From left to right: Total organic carbon and total carbon (wt%); molar C_org_/N ratio measured in bulk sediments; calcium, magnesium, chloride, and sulfate concentrations (μM) measured in the pore water; potential sulfate reduction rates (nmol × cm^-3^ day^-1^) obtained after 24 h incubation experiments; ammonium concentrations (μM) in the pore water measured by cell dialysis. **(B)** From left to right: Cell counts in log scale (log_10_ cells × cm^-3^); concentrations of extracellular DNA (gray dots) and total DNA (black squares) (μg × g wet sediment^-1^), with distance between the two curves corresponding to intracellular DNA concentrations; Shannon index established from bacterial and archaeal DGGE gel features, with eDNA (gray dots) and iDNA (black squares) displayed separately; range-weighted richness index for bacterial DNA (square) and archaeal DNA (dots), with eDNA (gray) and iDNA (black) displayed separately.

Sodium (Na^+^), calcium (Ca^2+^), and magnesium (Mg^2+^) were the only cations we were able to detect in the pore water by IC, only Ca^2+^ and Mg^2+^ were quantified. Ca^2+^ and Mg^2+^ profiles (**Figure 3A**) did not show much variability with depth, concentrations were around 25 μM and 130 μM for Ca^2+^ and Mg^2+^, respectively. The intermediate site displays one single significant peak at about 20 cm depth. At the deep site, Mg^2+^ values tend to increase slightly with depth to values around 150 μM. NH_4_^+^ concentrations in the uppermost pore water sample were similar at all three sites (ca. 22 μM) and showed a general increase with depth, the gradient became increasingly steeper from the shallow to the deep site (**Figure 3A**), leading to concentrations of 40, 60, and 130 μM at the bottom ends of the cores, respectively. Similarly, dissolved Fe^2+^ concentrations increased from the shallow to the deep site and with sediment depth, with values ranging from 0, 5, and 35 μM in surface sediment to 10, 20, and 50 μM at the bottom of the core, respectively. The Fe^2+^ profile of the deep site exhibits a slight excursion to lower values in the upper 10 cm of sediment, below this depth values increase constantly like at the other sites.

Chloride (Cl^-^) and sulfate (SO_4_^2-^) were the main inorganic anions in the pore water. Nitrate, nitrite, and phosphate were below detection limit. Profiles for Cl^-^ (**Figure 3A**) displayed constant concentrations around 40 to 20 μM with little difference between sites although with a general decrease with increasing water depth. Some minor peaks up to 60 μM could be observed in the upper 15 cm at all sites. SO_4_^2-^ concentrations were mainly in the single μM range and often close to the quantification (5 μM) and detection (2 μM) limits. At the shallow site, concentrations were generally higher, with values about 20 μM in the uppermost 4 cm, but decreasing gradually to values between 2 and 5 μM at the base of the core. At the intermediate site, sulfate was detectable at all depths but the measurements were close to or below the quantification limit, except for two peaks of 40 and 15 μM at 0.5 and 22.5 cm depth, respectively. At the deep site, all SO_4_^2-^ concentrations were around the detection limit.

Respective ion concentrations measured directly above the water–sediment interface in cores from the shallow, intermediate, and deep site are given in **Table 1**.

**Table 1:** Bottom water geochemistry of Lake Towuti.

	Geochemistry of bottom waters (μM)						
Site	Depth (m)	Ca^2+^	Mg^2+^	Cl^-^	SO_4_^2-^	NH_4_^+^
Shallow	60	28.6 ± 6.1	145.4 ± 8.9	37.3 ± 0.9	20.2 ± 0.7	<6
Intermediate	153	25.3 ± 0.1	134.8 ± 7.0	38.4 ± 1.9	11.9 ± 0.4	20.3 ± 0.8
Deep	200	23.8 ± 2.6	148.7 ± 4.3	20.5 ± 0.5	11.8 ± 0.2	13.8 ± 0.2

*Water samples were retrieved at the water–sediment interface in cores from the shallow, intermediate and deep site and measured by ion chromatography. Values are displayed in [μM] followed by one standard deviation*.

### Potential Sulfate Reduction Rates

Potential rates of sulfate reduction and their depth distribution (**Figure 3A**) differed significantly between sites. At the shallow site, we found maximum rates of ca. 7 nmol cm^-3^ day^-1^ within the upper 10 cm of sediment. Below this depth, SRR decreased sharply to ca. 2 nmol cm^-3^ day^-1^. The intermediate site displayed two peaks of elevated SRR of ca. 11 and 8 nmol cm^-3^ day^-1^ at 2 and 22 cm depth which corresponds to anomalous peaks in sulfate concentrations; at all other depths, rates were minimal <2 nmol cm^-3^ day^-1^. At the deep site, rates were generally lower than at the other sites (1 to maximum 3 nmol cm^-3^ day^-1^). Altogether, the measurements demonstrated that viable sulfate-reducing bacteria (SRB) were present at all three sites, but were more active at the shallow site. The results also showed a general decrease in SRR with increasing water depth due to decreasing bottom water SO_4_^2-^ concentrations and hence lower diffusive fluxes of sulfate into the sediment. Because SO_4_^2-^ concentrations were in many cases barely detectable (>2 μM) and well below the lower limit of quantification (5 μM), the absolute values of the potential SRR have to be carefully considered. We also acknowledge that our measured rates may differ from those on-site due to some biologically unavailable background SO_4_^2-^ (Roden and Tuttle, 1993; Roden and Wetzel, 1996). However, the data clearly show that the sulfate pool is turned over within days, implying strong internal recycling. In the absence of available SO_4_^2-^ in the pore water, the necessity to turn to reduced sulfur compounds also implies that SO_4_^2-^ reduction processes are slowed down.

### Organic Matter

TOC values (**Figure 3B**) increased from the shallow to the deep site. The shallow site had TOC contents around 2% in the upper few centimeters with a subsequent increase by about 1% and constant values over the rest of the core. The intermediate site exhibited oscillating values between 4.5 and 3.5% in the uppermost 10 cm, continuing with lower and more constant values of ca. 3–3.5% down to the base of the record. At the deep site, TOC concentrations decreased gradually from 4.5% in the uppermost section to 3% at the base of the core. In parallel to these results, TC peaks around 5 cm depth at both the intermediate and deep site indicated the likely presence of authigenic carbonates (**Figure 3B**).

The C_org_/N ratio profiles resembled those of TOC, with values generally increasing from the shallow site to the deep site. Overall, values increased gradually from 8.5 to 12.5 at the shallow site and varied in between 10.5 and 12.5 at the intermediate site. At the deep site, the ratios ranged from 10.5 to 15.5 in the uppermost samples and gradually decrease to 11.5 in the lower part. Altogether, TOC and C_org_/N ratio profiles appear to reflect the different bottom water oxygenation conditions at the three sites as values generally increase with water depth.

### Total Cell Counts

Total cell counts (**Figure 3B**) were much higher at the shallow site than at the other sites. At all three sites, numbers declined steeply in the upper 5 cm, followed by a more gradual decrease over the remainder of the core. Compared to the other two sites, cell concentrations at the shallow site were up to 31 times higher in the 0–1 cm depth interval (i.e., log_10_ = 9.6, 8.4, and 8.1) and still up to three times higher at the base of the cores (i.e., log_10_ = 8.2, 7.8, and 7.7). The profiles from the intermediate and deep site were very similar, although values were generally lower at the deep site.

### Intracellular and Extracellular DNA Concentrations

Concentrations of eDNA (**Figure 3B**) were very similar at all three sites, with concentrations around 0.6 μg g^-1^ in the uppermost layer, decreasing to ca. 0.2 μg g^-1^ at 10 cm depth and a final decline to minimum values (<0.1 μg g^-1^) at the bottom of the core. Total DNA (tDNA = eDNA + iDNA) concentration profiles mimic those of eDNA, with concentrations of 1.6, 1.2, 1.2 μg g^-1^ in the surface layer and 0.4, 0.2, and 0.3 μg g^-1^ in the bottom layer for the shallow, intermediate, and deep site, respectively. Results showed that concentrations of eDNA and iDNA display similar trends and that iDNA was the dominant fraction in total DNA.

In order to assess the recovery rate of cells and respective amount of extracted iDNA with this procedure, iDNA concentrations were divided by the corresponding cell densities of each sample and results reported in femtogram (fg) of DNA per cell. Averaged values per site were 1.0, 3.7, and 6.5 fg DNA cell^-1^ at the shallow, intermediate and deep site, respectively (data not shown). Published values range from 1.6 to 8.9 fg DNA cell^-1^ (Bakken and Olsen, 1989; Button and Robertson, 2001). In nearby Lake Matano, phosphorus limitation resulted in reduced RNA concentrations per cell (Yao et al., 2016). Similarly in Lake Towuti, the variable sorption capacity of the sediment could influence P availability and DNA concentration per cell. Concerning the presence of viral DNA (Torti et al., 2015), protein capsids are not quantified as DNA with a Qubit 2.0 fluorometer. Moreover, sediment particles also strongly adsorb virions and multiple studies of freshwater sediments showed a low impact of viruses on bacterial mortality (Filippini and Middelboe, 2007 and references therein). We acknowledge that viral DNA should be found in our extracts, albeit as a minor fraction as we did not use a specifically adapted extraction protocol necessary to lyse the capsids (Danovaro and Middelboe, 2010).

### Bacterial and Archaeal Fingerprinting

DGGE provided an assessment of the relative preservation of eDNA at the three sites along with a first fingerprinting of bacterial and archaeal diversity associated with both eDNA and iDNA fractions. In general, eDNA gel features faded with increasing sample depth and led to the transformation of clear bands into smears and further disappearance (Supplementary Material). At all three sites, Shannon indices of bacterial eDNA (**Figure 3B**) decreased almost linearly with depth, whereas those of bacterial iDNA display different trends at each site. Values increased within the upper part of the core before gradually decreasing at the shallow site, decreased linearly at the intermediate site and sharply dropped in the lower half of the core at the deep site. Values of archaeal eDNA (**Figure 3B**) rapidly declined in the upper 20 cm and stayed minimal below that depth at the shallow site, showed high scatter and little decrease at the intermediate site and dropped at ca. 20 cm followed by constant values at the deep site. Shannon values of archaeal iDNA were similar for each site, with fluctuations in upper sediments and gradual decrease in lower sediments, although the intermediate site generally displayed lower values. Richness indices (Supplementary Material) clearly demonstrated the predominance of Bacteria over Archaea.

### Phylogeny of Sequenced DGGE Fragments

Sequences of eDNA were mainly representative of aerobic and anaerobic heterotrophs, with taxa identified as Actinobacteria, Nitrospirae, Chloroflexi, and Thermoplasmatales (Supplementary Material). Taking into account the local conditions of the sediment, these sequences were likely related to microorganisms evolving in the water column and preserved as eDNA in the sediment. However, Actinobacteria are known ubiquists in soils, aquatic habitats and sediments and could also reflect a terrestrial origin arising from eroded soils transported to the lake. We considered eDNA sequences clustering among iDNA-related taxa to derive from cell lysis in the sediment (e.g., Betaproteobacteria, Deltaproteobacteria, Firmicutes). Additionally, planktonic species in the bottom waters could become benthic at the water–sediment interface before being buried and could, thus, also be preserved as iDNA.

**FIGURE 4:**
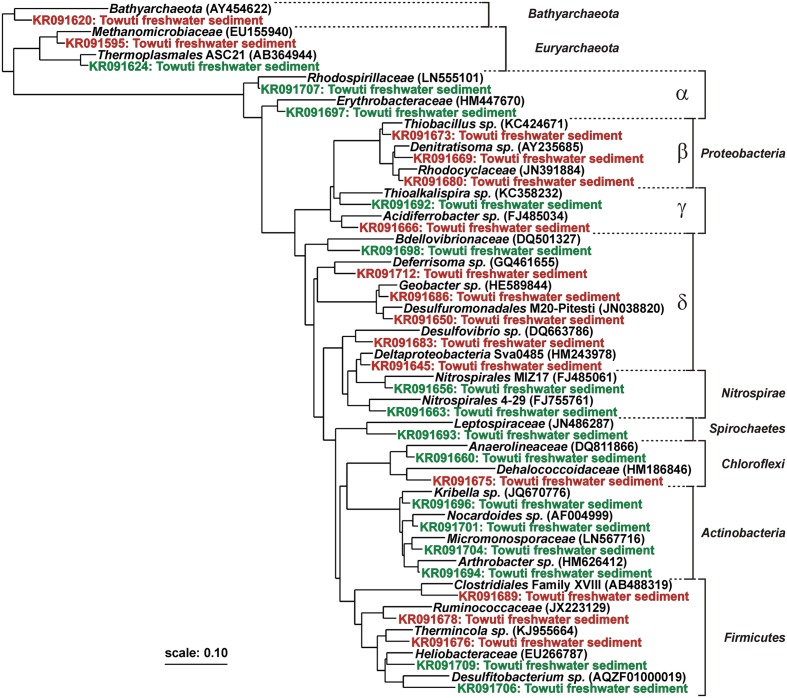
**Phylogenetic tree established for intracellular and extracellular DNA fragments obtained from DGGE gels.** Among 131 sequences, 30 were selected as representative taxa to build a maximum-likelihood RAxML tree. Extracellular DNA sequences (green) are indicative of aerobic and anaerobic heterotrophs, among which some can be assigned to the lake oxic (e.g., *Nitrospirae*, *Actinobacteria*) and anoxic water column (e.g., *Chloroflexi*, *Firmicutes*). Intracellular DNA sequences (red) are closely affiliated with known sulfate and iron reducers (e.g., *Deltaproteobacteria*, *Firmicutes*). Concomitant presence of taxa related to *Clostridiales* and *Methanomicrobiales* indicates a potential for heterotrophic production of fermentative hydrogen and methane. Boldface types signify database references with sequence accession numbers in parentheses.

Taxa identified from iDNA fragments were mostly related to Beta- and Deltaproteobacteria, Firmicutes, and Methanomicrobiales (**Figure 4** and Supplementary Material). Sequences of Betaproteobacteria plotted among Rhodocyclaceae with closest cultivated taxa represented by *Thiobacillus* and *Denitratisoma*. Sequences of Deltaproteobacteria were closest to *Desulfovibrio*, *Desulfuromonas*, *Deferrisoma*, *Geobacter*, and candidate order Sva0485. Together with the potential SRR, these sequences provided evidence for the presence of sedimentary SRB along with microorganisms capable of dissimilatory sulfur and ferric iron reduction at all three sites. Sequences of Firmicutes related to *Thermincola* supported the presence of iron-reducing bacteria. Finally, taxa among Ruminococcaceae and Clostridiales were representative of heterotrophic anaerobes, whose concomitance with Methanomicrobiales indicated that fermentative hydrogen can be produced via ferredoxin reduction and used during methanogenesis (Hallenbeck, 2009).

## Discussion

### Water Column Conditions and Organic Matter Sedimentation

Varying oxygenation levels in the water column, especially at the water–sediment interface are expected to lead to differences in production, degradation, and burial of OM in sediments at the three sites (Katsev and Crowe, 2015 and references therein). Chlorophyll *a* concentrations are highest around 50 m water depth, while transmission profiles indicate suspended matter close to the oxycline at 130 m water depth (**Figure 2**), pointing toward a redox interface similar to the one observed in the water column of nearby Lake Matano, although less steep (Crowe et al., 2008a). At the intermediate site, a long-term shift in the oxycline was recorded during the mid-Holocene in the form of elevated ferric iron and siderite (i.e., FeCO_3_) concentrations (Costa et al., 2015). Due to the low sedimentation rates (i.e., 2 mm year^-1^; Vogel et al., 2015), redox fluctuations would be hard to detect at our sampling resolution.

TOC contents indicate increased OM burial from the shallow to the deep site (**Figure 3A**), associated with a shift toward more recalcitrant OM composition (i.e., higher C_org_/N ratio). Although degradation processes in an anoxic water column are generally slower (Katsev and Crowe, 2015), iron reduction below the oxycline can foster desorption of labile OM and microbial uptake of nitrogen and phosphorus (Zegeye et al., 2012), thereby increasing carbon burial linked to bacterial production. Remineralization of organic compounds leads to the production of NH_4_^+^, which can be oxidized or accumulate, depending on the presence of suitable reactants in the water column. NH_4_^+^ concentrations in bottom waters (**Table 1**) indicate that degradation of sinking particulate OM is indeed continuous in the water column, leading to the accumulation of less reactive organic material at the deep site (Amon and Benner, 1996). This interpretation is also consistent with the diverse taxa identified from our eDNA sequences, which corresponded to both aerobic and anaerobic heterotrophs, namely Actinobacteria, Nitrospirae, Chloroflexi, and Thermoplasmatales, and secondarily Alpha- and Gammaproteobacteria (**Figure 4**; and Supplementary Material). However, final discrimination of eDNA sources cannot be achieved. For example, Actinobacteria could originate from soils eroded from the catchment, transported to the lake and preserved within refractory OM. Although we did not detect any photoautotrophs, certain elements can still be assigned to planktonic assemblages coming from the epilimnion (e.g., Nitrospirales) and hypolimnion (e.g., Anaerolineales). Our interpretation is that labile OM from primary producers is rapidly degraded by heterotrophic species that are active in the water column, resulting in the preferential preservation of heterotrophic over phototrophic sequences in the eDNA (Vuillemin et al., 2016). This is also consistent with the transmission decrease at the oxycline (i.e., 130 m), which we interpret as iron reduction leading to desorption of phosphorus compounds and thereby promoting anaerobic heterotrophic processes. Data from Lake Matano show that dissolution of particulate Fe^3+^ occurs at and below the pycnocline along with the liberation of phosphorus and concomitant production of methane and ammonia (Crowe et al., 2008b; Katsev et al., 2010).

### Pore Water Geochemistry

High Ca^2+^ and Mg^2+^ concentrations in pore waters (**Figure 3A**) likely reflect continued weathering of mafic/ultramafic minerals derived from the catchment of the Malili Lake system (Kadarusman et al., 2004). Although fairly constant, variations in these profiles could possibly imply precipitation and/or dissolution of authigenic carbonates (i.e., Ca–Mg–FeCO_3_) within the sediment. At the intermediate and deep site, the presence of authigenic carbonates can be inferred from peaks in TC profiles. The diagenetic sequence known for freshwater sediments in relation to pore water geochemistry (i.e., Ca–Mg siderite, calcite, ankerite, dolomite) suggests the formation of early diagenetic siderite (Berner, 1980; Matsumoto and Iijima, 1981), which is consistent with the decline in dissolved Fe^2+^ concentrations at the deep site. Lake Matano and Lake Towuti are both iron-rich but also extremely low-sulfate environments, far lower than most aquatic environments studied to date (Crowe et al., 2014). Moreover, microbial reduction processes in the water column likely take place at or directly below the oxycline, as shown for SO_4_^2-^ concentrations in Lake Matano, which decrease drastically along with a rapid increase of dissolved Fe^2+^ (Crowe et al., 2008a). In Lake Towuti, SO_4_^2-^ concentrations measured at the water/sediment interface decreased from the shallow to the deep site (**Table 1**), supporting our assumption that sulfate reduction already takes place in the anoxic bottom water of the intermediate and deep site. As a result pore water SO_4_^2-^ concentrations in the uppermost sediment samples decreased toward the deep site and were rapidly depleted with sediment depth at all sites. Pore water Fe^2+^ concentrations show the opposite trend, increasing from the shallow to the deep site and with core depth, showing an increasingly steeper gradient between sites with maximum values around 45 μM (**Figure 3A**). The production, consumption, and dynamics of NH_4_^+^ in bottom waters seemed to differ between the three sites as indicated by their respective concentrations (i.e., <6, 20, and 13 μM) and the very similar NH_4_^+^ concentrations in the uppermost sediment sample at all three sites (ca. 25 μM). The NH_4_^+^ profile of the deep site is indeed the only one that clearly indicates NH_4_^+^ diffusion out of the sediment into the bottom waters. Whereas nitrification could take place in the oxic bottom waters of the shallow site, the potential accumulation of NH_4_^+^ in the anoxic bottom waters of the two other sites remains unclear.

### Sediment Microorganisms and Extracellular DNA Remineralization

Several studies showed that bacterial abundance and diversity in lake sediments correlate with environmental parameters such as salinity, pH, OM content, and sediment depth (Nam et al., 2008; Zeng et al., 2009; Borsodi et al., 2012), and can also reflect climatic variations due to forcing of conditions during sediment deposition (Dong et al., 2010; Vuillemin et al., 2013, 2014). Lake Towuti’s surface sediments are characterized by comparatively high cell densities that decrease from the shallow to the deep site. At all sites, cell counts decrease by one order of magnitude over the uppermost 5 cm below the water–sediment interface and remain more or less constant over the rest of the core (**Figure 3B**). The corresponding iDNA concentrations follow a similar trend, whereas Shannon indices show more variability between the three sites. Potential SRR measurements with radiotracer (**Figure 3A**) further demonstrate that SRB are present and viable at all sites, but are more active at the shallow site, showing some correspondence to Shannon indices. We interpret these data as the positive response of bacterial populations to geochemical conditions corresponding with higher bottom water SO_4_^2-^ concentrations and increased burial of labile OM and reactive ferric iron into the sediment.

With regard to recording of past lake conditions by eDNA, water temperatures of Lake Towuti are approximately 28°C throughout the year, which is rather unfavorable for eDNA preservation (Lindahl, 1993; Renshaw et al., 2015). Although sorption capacities in the sediment were expected to vary between the three sampling sites, eDNA distribution patterns are similar, displaying a rapid decrease in the uppermost 5 cm followed by constant but low concentrations over the rest of the cores (**Figure 3B**). The decline of eDNA Shannon indices also indicates a gradual loss of genetic information associated with the degradation and shortening of eDNA sequences over time (Corinaldesi et al., 2008). Such decrease as a function of sediment depth can be attributed to a combination of sediment sorption capacity, microbial uptake, and degradation as well as rates of cell lysis. We argue that the observed lower eDNA concentrations result from an overall decrease in metabolic activity and cell lysis rate, along with the immediate degradation of the free eDNA fraction resulting in diminishing Shannon values. In the long-term, eDNA preservation would greatly depend on metabolic turnover rates and its potential stabilization by ferric mineral phases (Glasauer et al., 2003; Pinchuk et al., 2008). In this context, preliminary results indicate that reactive ferric iron persists down to 15 m sediment depth at concentrations up to nearly 2 wt% (Simister et al., 2016).

### Phylogeny of Intracellular DNA and Presumed Metabolic Processes

Microbial fingerprinting (**Figure 4**; and Supplementary Material) revealed the presence of microorganisms related to taxa commonly known for iron and sulfate reduction as well as methanogenesis, indicating that the autochthonous microbial communities have the metabolic capacity for these processes. Among Proteobacteria, certain taxa were affiliated with *Denitratisoma* (Fahrbach et al., 2006), *Thiobacillus* (Schedel and Trüper, 1980; Haaijer et al., 2006), and Acidiferrobacter (Hallberg et al., 2011), indicating putative capacity for both lithotrophic and organotrophic processes driven through use of reduced sulfur and iron as electron donors. However, these Beta- and Gammaproteobacteria typically exhibit metabolic versatility with the capacity to shift between different modes of facultative metabolisms (Masters and Madigan, 1983; Hiraishi and Hoshino, 1984; Ferguson et al., 1987), making it impossible to assess metabolism from taxonomic information alone. This could also indicate that these taxa are planktonic in the bottom waters, then benthic at the water–sediment interface until burial.

Interestingly, sequences of Deltaproteobacteria at the shallow and intermediate site included taxa assigned to the candidate order Sva0485 and *Desulfovibrio*, which are often reported as part of SRB consortia (Kleindienst et al., 2014; Bar-Or et al., 2015). These sequences were correspondingly identified in sediments with the highest measured SRR. In addition, detection of *Desulfuromonas* M20-Pitesti, *Deferrisoma* and *Geobacter*-related sequences may indicate that the metabolic capacity to reduce sulfur and iron is evenly distributed across all three sites (Slobodkina et al., 2012; Greene, 2014). DGGE fragments affiliated with *Thermincola* also point at a metabolic potential for iron reduction (Zavarzina et al., 2007). In addition, taxa related to Ruminococcaceae and Clostridiales were representative of heterotrophic anaerobes that can produce fermentative hydrogen via ferredoxin (i.e., Fe_2_S_2_ protein cluster) reduction (Hallenbeck, 2009). Their concomitance with Methanomicrobiales suggests that H_2_/CO_2_ reduction is a likely pathway for methane production (Thauer et al., 2008; Kaster et al., 2009), although formate and alcohols could also be used (Ohren, 2014).

Together, our measurements of potential sulfate reduction and our findings of microbial taxa commonly involved in microbial sulfate and iron reduction suggest that ferruginous sediments support microorganisms that degrade OM via sulfate reduction, in spite of the extremely low sulfate concentrations, in collaboration with diverse iron-reducing bacteria, fermenters, and methanogens. Seemingly, such complementary metabolisms imply desorption of OM following iron reduction and its remineralization to methane. The low pore water SO_4_^2-^ concentrations (single μM) and the relatively high potential SRR (single to tens of nmol cm^-3^ day^-1^) also demonstrate that the sulfate pool is turned over within days. This suggests that reoxidation of reduced sulfur compounds occurs through a cryptic S-cycle driven by iron (Norði et al., 2013; Hansel et al., 2015) in order to maintain these high SRR. A likely mechanism for such recycling is the disproportionation of elemental sulfur linked to ferric iron reduction (Thamdrup et al., 1993; Holmkvist et al., 2011). Moreover, unless methanogens are being outcompeted by iron reducers (Roden and Wetzel, 1996), the persistence of ferric iron in deeper sediment could be seen as an indicator for processes involved in anaerobic oxidation of methane (Hallam et al., 2004).

## Conclusion

Stratification of Lake Towuti’s water column gave rise to different biogeochemical conditions in deeper parts of the water column and at the water–sediment interface at the three study sites. Respiration processes led to the gradual depletion of electron acceptors with increasing water depth, with microbial Fe^3+^ and SO_4_^2-^ reduction occurring below the oxycline. As a result of microbial uptake of nitrogen and phosphorus, carbon burial increased with water depth while NH_4_^+^ accumulated in anoxic bottom waters. Sediments at the shallow site exhibited more labile OM as well as higher pore water SO_4_^2-^ concentrations and, consequently, harbored the highest cell densities and potential SRR. Retrieved eDNA sequences confirmed the role of microbial degradation within the water column, with some aerobic and anaerobic heterotrophic elements potentially linked to the epilimnion and hypolimnion. Nevertheless, eDNA was substantially degraded in the uppermost sediment layers at all three sites, leading to the gradual loss of genetic information. Fingerprinting of iDNA revealed taxa common in SRB consortia, along with known iron reducers and methanogens at all sites. Our results attest that Lake Towuti’s sediments support microorganisms displaying complementary metabolic capabilities related to sulfur, iron and methane cycling. Relatively high SRR could be maintained in these ferruginous sediments through a cryptic sulfur cycle driven by iron reduction. The related loss of the sedimentary OM sorption capacity over time would then promote OM remineralization to methane. However, ferric iron phases may persist in deeper sediment layers, questioning the availability of organic substrates rather than that of reactive iron. To conclude whether they be related to climate or in-lake processes, redox changes in the water column appear to lead to variable burial of OM, electron acceptors and reactive metal species in the sediments. Regarding the entire lacustrine record, a long-term shift to more oxic conditions would lead to persistence of electron acceptors in deeper sediments and could promote metabolic activity by the subsurface biosphere.

## Author Contributions

AV performed DNA extractions, DGGE procedure, genetic and image analyses, designed the figures, and led the writing of the present manuscript. AF sampled during field campaign, performed geochemical analyses and cell counts. MA designed and supervised DNA extractions and genetic analyses. SAC sampled during field campaign and conducted the CTD cast measurements. CH and SN fulfilled the research permit procedure and sampled during field campaign. DW provided important financial and technical support and supervised genetic analyses. JK designed the study, sampled during field campaign, supervised geochemical analyses and cell counts. All authors have taken part in the manuscript revisions and agreed with its scientific content.

## Conflict of Interest Statement

The authors declare that the research was conducted in the absence of any commercial or financial relationships that could be construed as a potential conflict of interest.
