# Supplementary material for: Geomicrobiological Features of Ferruginous Sediments from Lake Towuti, Indonesia
Source: Front Microbiol. 2016 Jun 30;7:1007. doi: 10.3389/fmicb.2016.01007 (PMC4928248; doi:10.3389/fmicb.2016.01007)
Supplement: Supplementary file 1 [file Data_Sheet_1.PDF]

frontiers

Supplementary Material

Geomicrobiological features of ferruginous sediments from Lake Towuti, Indonesia

Aurèle Vuillemin, André Friese, Mashal Alawi, Cynthia Henny, Sulung Nomosatryo, Dirk Wagner, Sean A. Crowe, Jens Kallmeyer*

*** Correspondence:** kallm@gfz-potsdam.de

1. Supplementary Figures

1) RGB values extraction

Scanelines are positioned on the samples and spectra extracted from the image based on simultaneous total red-green-blue (RGB) channels. For a reverse image, signal quantification is scaled from white (R:0/G:0/B:0) to black (R:255/G:255/B:255) and normalized. If DGGE images are in binary mode, the extraction of one channel is enough.

2) Peak comparison between samples

This step allows for checking sample alignment between lanes as migration of DNA in the gel is not always uniform. It also provides direct comparison in terms of gain or loss of intensity for one same band between samples.

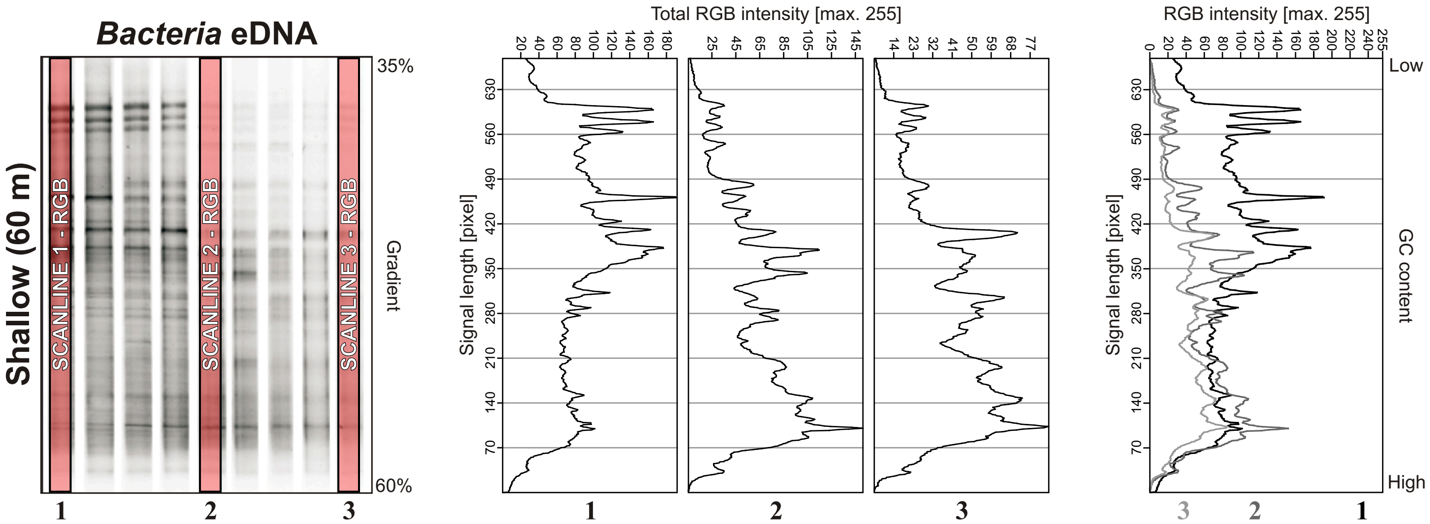

3) Peak and band identification

Scaneline images and their spectra are viewed simultaneously in order to authenticate peaks corresponding to bands. Smears can indicate the presence of residual bands and caution is required if using the automated function of peak extraction.

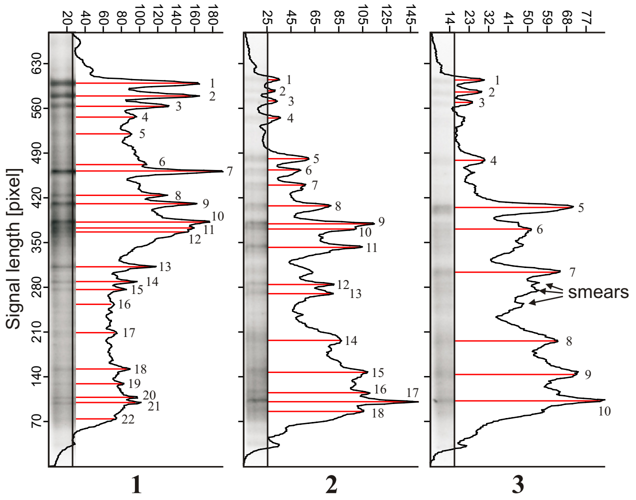

decreasing intensities
decreasing number of bands
preserved bands in the bottom part (GC-rich)

4) Band intensities and gradient length

Spectral intensity (X axis) and pixel position (Y axis) are exported as .txt file. Band intensities and length ratios between total signal and band pattern are used in the calculation of the Shannon and Richness indices, respectively.

Peak no.	RGB Intensity	RGB Intensity	RGB Intensity
1	165.3	32.92	30.61
2	164.41	30.38	39.91
3	130.36	31.28	25.78
4	95.71	33.65	31.63
5	91.12	54.82	74.11
6	108.59	49.35	53.72
7	193.19	52.04	66.68
8	100.85	70.64	64.42
9	131.51	104.23	72.83
10	163.88	91.98	85.55
11	179.55	95.44	
12	163.28	74.76	
13	120.5	93.77	
14	122.38	83.61	
15	99.98	102.73	
16	87.8	108.15	
17	73.92	145.02	
18	75.74	102.07	
19	90.17		
20	83.14		
21	98.69		
22	102.38		
**TOTAL**	**2642.45**	**1336.84**	**545.24**
	1	2	3

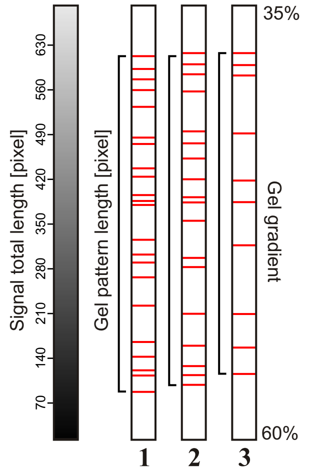

Shannon

$$-\sum (ni/N) \times \log (ni/N)$$

ni = each peak intensity
N = sum of all peak intensities

Richness

$$N^2 \times D_g$$

N = total band number
D_g = pattern gradient %

Figure S1. Signal extraction from DGGE gels using the image software Strati-Signal. Scanlines extract values from selected channels, presently red (R) green (G) and blue (B) simultaneously, transforming image gel patterns into quantitative spectra (**1**, **2**). Peaks corresponding with bands are identifed automatically or manually (**3**). Respective positions and intensities are exported into a .txt file and used for statistical purposes (**4**). The present example shows that intensity and number of bands decrease in gel patterns with sample depth . Smears in spectra are indicative of residual bands not always visible on gel images.

**Shallow (60 m)**				
	Bacteria		***Archaea***	
**Depth**	**iDNA**	**eDNA**	**iDNA**	**eDNA**
[cm]	[bd nb]	[bd nb]	[bd nb]	[bd nb]
0.5	23	22	16	21
1.5	27	20	17	17
5.5	30	20	11	15
7.5	32	17	13	16
11	31	16	11	7
15	21	12	7	5
19	21	8	7	4
22.5	22	7	7	4
27.5	22	6	5	4

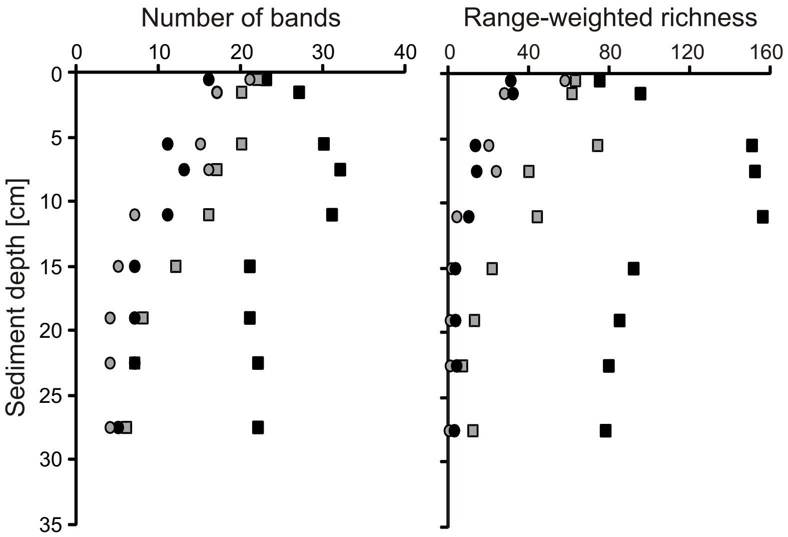

**Intermediate (153 m)**				
	Bacteria		***Archaea***	
Depth	**iDNA**	**eDNA**	**iDNA**	**eDNA**
[cm]	[bd nb]	[bd nb]	[bd nb]	[bd nb]
1.5	32	29	9	11
4.5	29	23	10	16
7.5	30	23	6	8
11	(16)	19	8	9
15	25	12	7	16
19	23	11	5	6
22.5	23	9	4	9
27.5	23	12	5	9
32.5	19	10	4	8

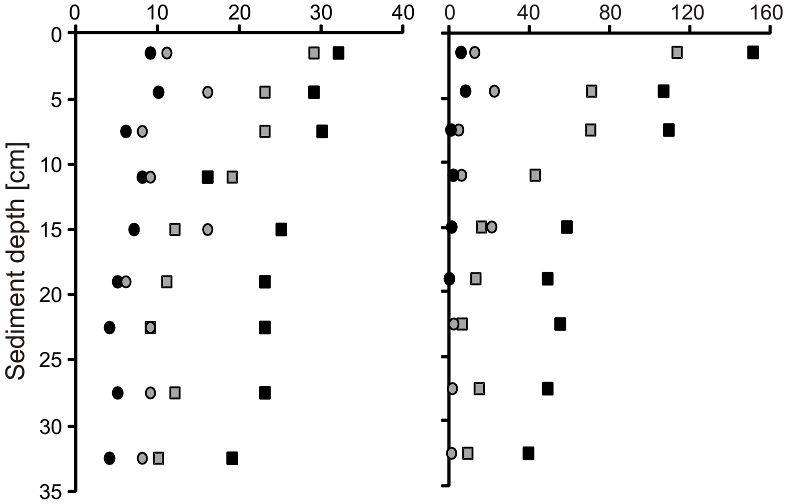

**Deep (200 m)**				
	Bacteria		***Archaea***	
Depth	**iDNA**	**eDNA**	**iDNA**	**eDNA**
[cm]	[bd nb]	[bd nb]	[bd nb]	[bd nb]
1.5	30	28	19	10
4.5	32	25	14	13
7.5	24	21	9	7
11	22	18	10	12
15	22	19	9	7
19	11	21	11	4
22.5	11	12	9	4
27.5	10	14	7	4
32.5	14	9	5	5

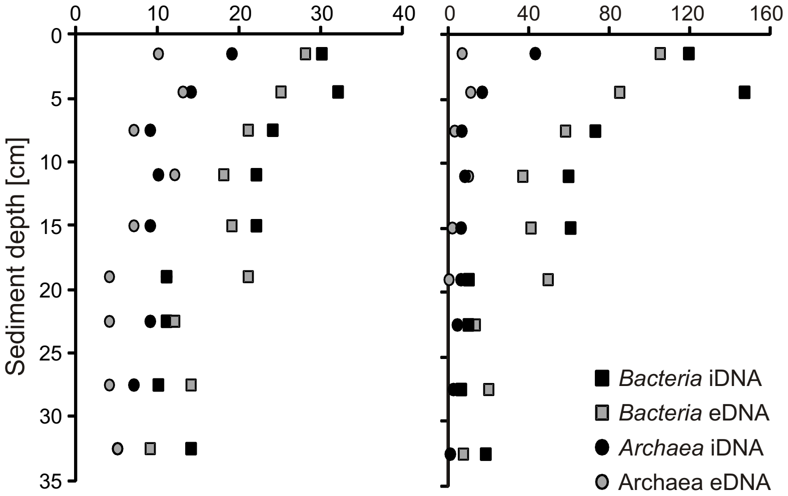

Figure S2. Summary of the number of bands extracted per sample. Tables list the number of bands counted for each sample based on their respective DGGE gel patterns (left), followed by band numbers plotted against sample depth for the three sites (center) and range-weighted Richness calculated as described in supplementary figure 1 (right).

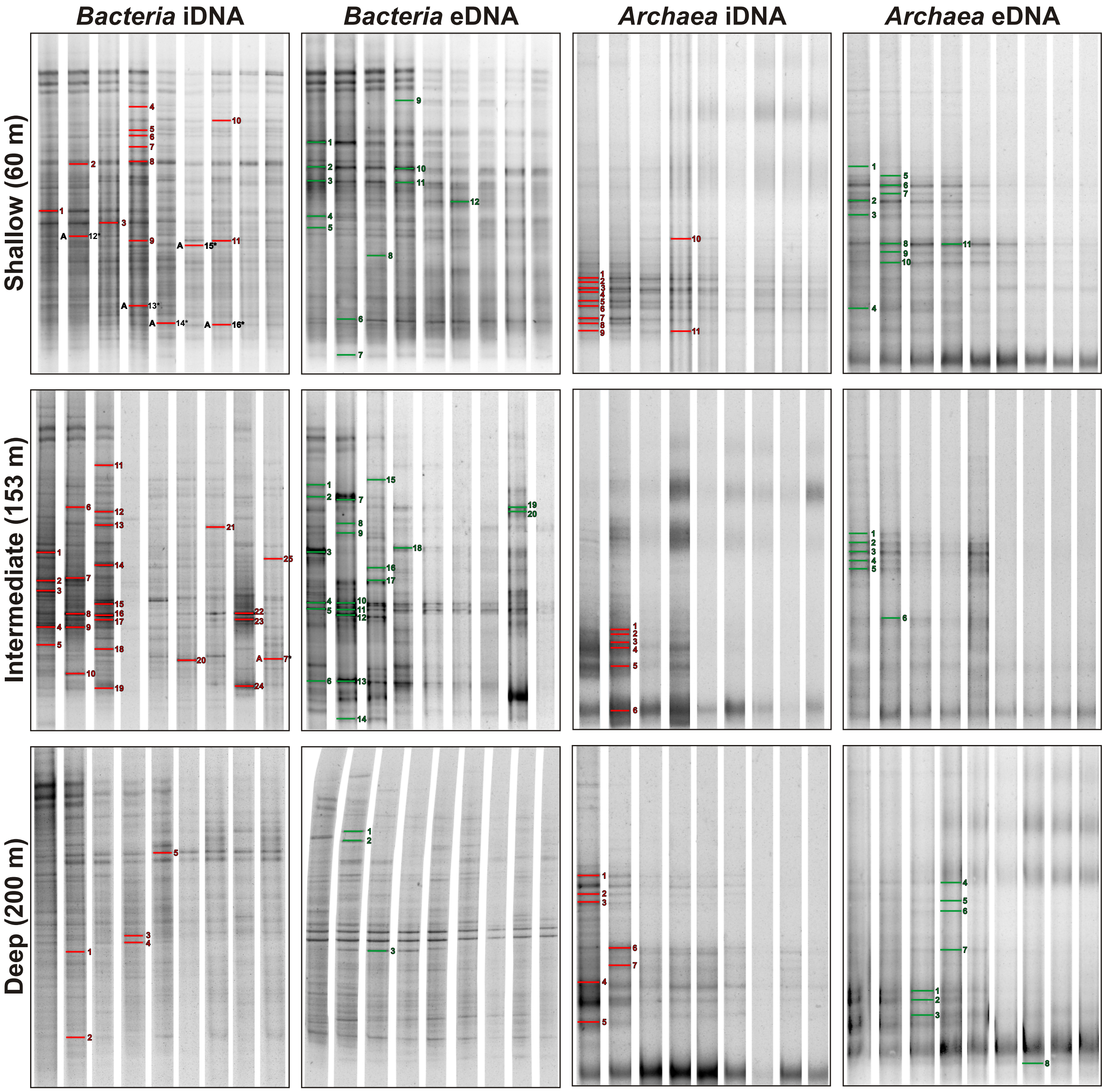

Figure S3. Pictures of DGGE gels obtained for a gradient from 35 to 60 %. Each DGGE gel was processed via image analysis (supplementary figure 1) and statistical indices calculated for every sample. Red and green bars highlight intracellular and extracellular bands that were picked up and sequenced. Band reference numbers are reported in the bacterial and archaeal phylogenetic trees.

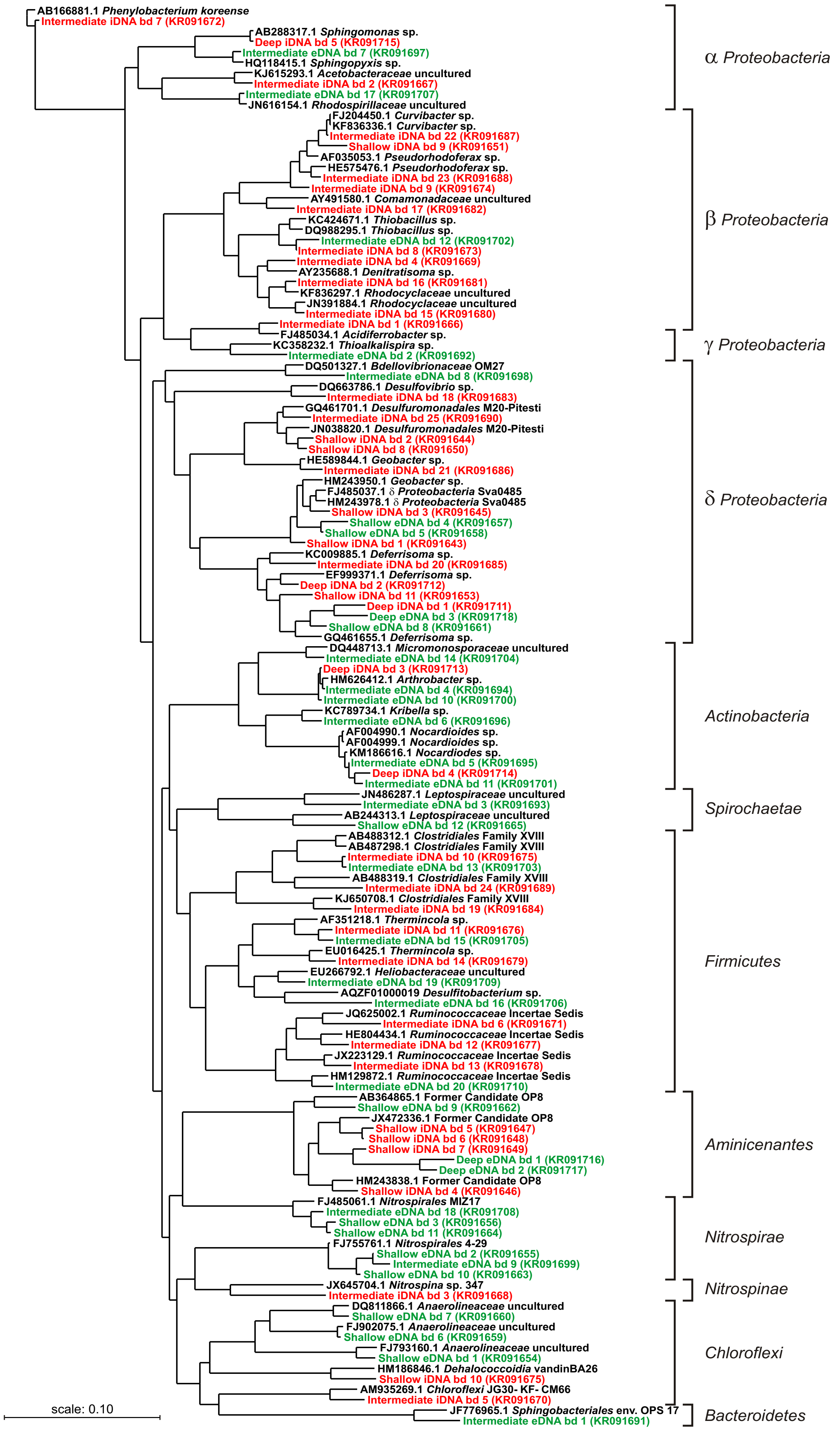

Figure S4. Phylogenetic tree established for bacterial DNA fragments (250 to 400 bp) obtained from DGGE gels. Extracellular (green) and intracellular (red) DNA sequences mainly cluster separately. Sequence interfingering may point at cell lysis as an additional source of extracellular DNA. Boldface types signify database references. Accession numbers for Lake Towuti sequences are indicated in parentheses.

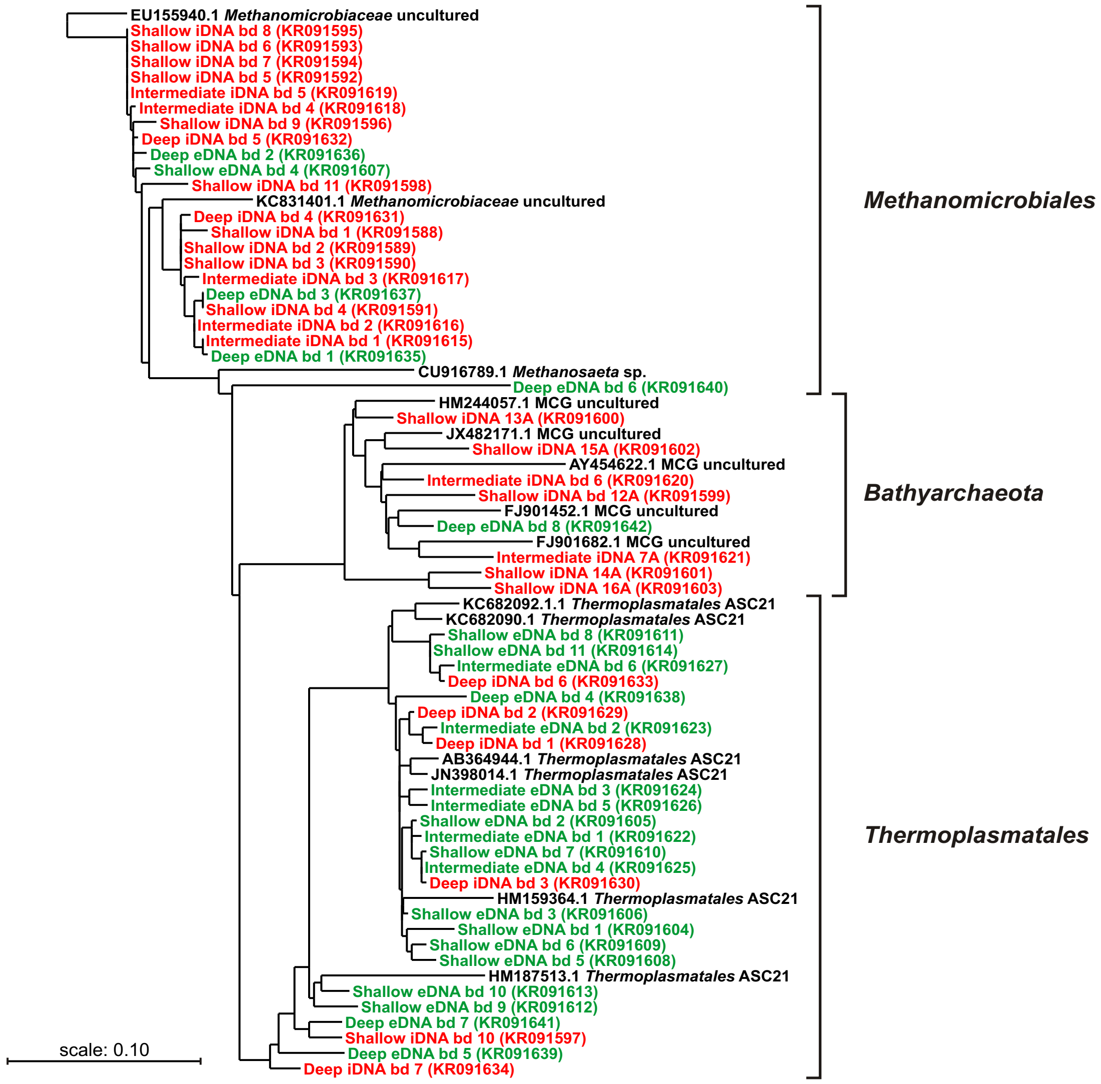

Figure S5. Phylogenetic tree established for archaeal DNA fragments (250 to 400 bp) obtained from DGGE gels. Most extracellular (green) and intracellular (red) DNA sequences cluster separately with little interfingering. *Thermoplasmatales* and *Methanomicrobiales* were respectively identified as eDNA and iDNA sequences, likely indicating that methanogenic populations in the water column differ from those in the sediment. Boldface types signify database references. Genbank accession numbers for Lake Towuti sequences are indicated in parentheses.
